# Supplementary material for: Vertebrate myosin 1d regulates left–right organizer morphogenesis and laterality
Source: Nat Commun. 2018 Aug 23;9:3381. doi: 10.1038/s41467-018-05866-2 (PMC6107537; doi:10.1038/s41467-018-05866-2)
Supplement: Supplementary file 1 — Supplementary Information [file 41467_2018_5866_MOESM1_ESM.pdf]

Vertebrate *myosin1d* regulates Left-Right Organizer morphogenesis and laterality

Manush Saydmohammed[1], *Hisato Yagi*[1], *Michael Calderon*[2], Madeline J. Clark[3], *Timothy Feinstein*[1], *Ming Sun*[2], *Donna B. Stolz*[2], *Simon C. Watkins*[2], *Jeffrey D. Amack*[3], *Cecilia W. Lo*[1], *Michael Tsang*[1]

[1]Department of Developmental Biology, University of Pittsburgh, 3501 5th Avenue, 15213

[2]Department of Cell Biology, University of Pittsburgh, 3500 Terrace Street, Pittsburgh, PA 15261

[3]Department of Cell and Developmental Biology, State University of New York Upstate Medical University, 750 East Adams Street, Syracuse, NY 13210

Supplementary Information

Supplementary Figure 1

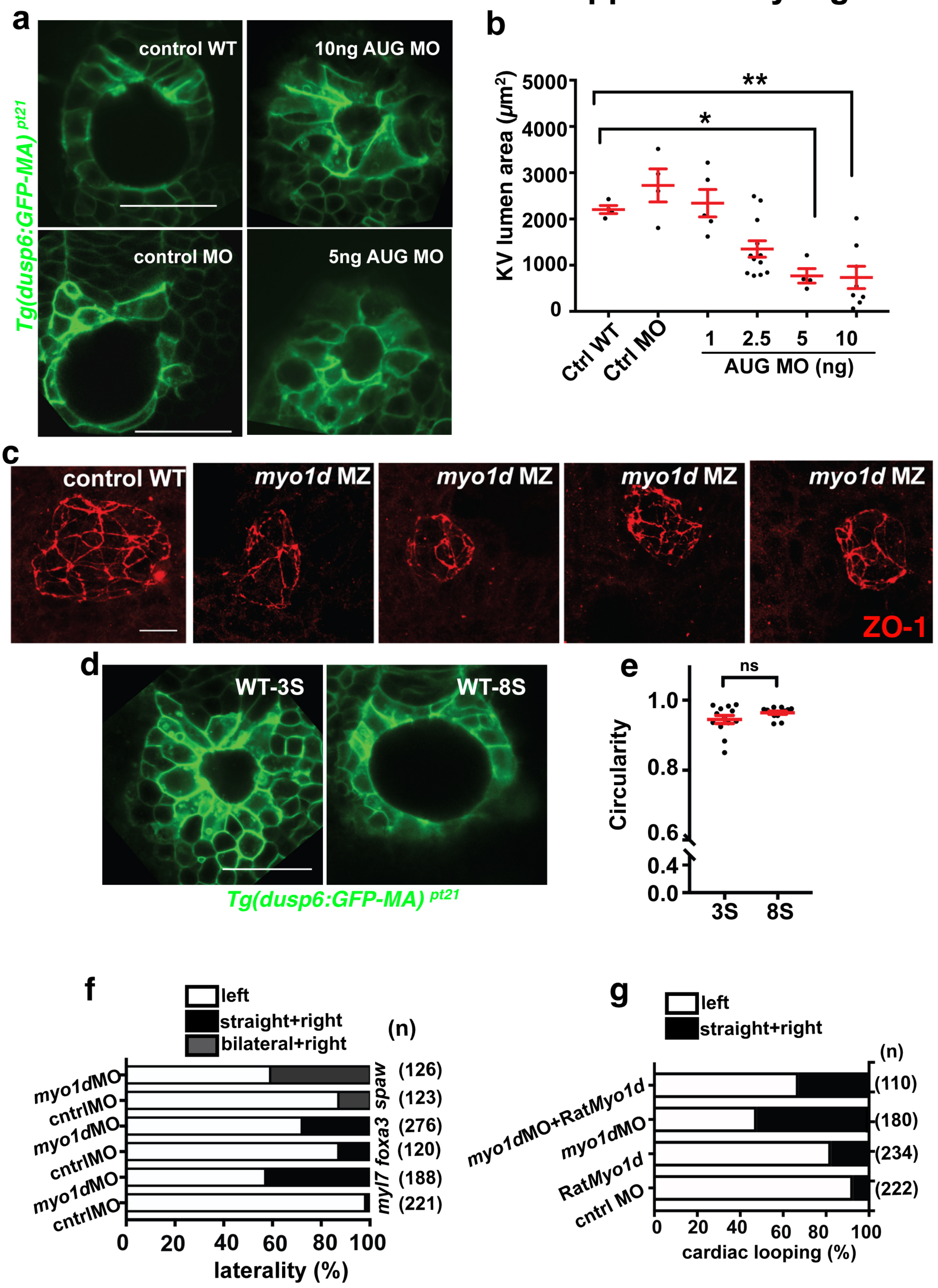

Supplementary Figure 1. *myo1d* is necessary for lumen morphogenesis of Kupffer's vesicle

a. Representative images showing KV lumen surface area with increasing doses of *myo1d* AUG MO injected into *Tg(dusp6:GFP-MA)* embryos.
b. Graph showing KV lumen formation was significantly decreased with increasing doses of *myo1d* AUG MO injected into embryos.
c. Loss of Myo1d leads to dysmorphic lumen formation. ZO1 staining marks apical surface in *myo1d* MZ embryos showing smaller or dysmorphic KV shape when compared to controls. scale bar-20μm.
d. Representative images of *Tg(dusp6:GFP-MA)* embryos showing KV formation at 3S and 8S stages used to calculate circularity.
e. Graph showing circularity of KVs from 3S and 8S embryos.
f. Laterality, expressed as percentage, from MO injected embryos. Embryos were fixed and probed using RNA in situ probes for *myl7, foxa3 and spaw*, and scored for defective looping pattern. Knockdown of Myo1d increased laterality defects.
g. Cardiac looping, expressed as percentage from MO injections and rescue with rat *Myo1d* mRNA. Injected embryos fixed and probed for *myl7* expression as a marker for cardiac looping. Knockdown of *myo1d* resulted in looping defects that was reduced with co-injection of rat *Myo1d* mRNA.

Statistical comparisons for the graphs were by ANOVA and post hoc analysis with Turkey's multiple range tests. Two sample comparisons were from unpaired t test. * $p<0.05$, ** $p<0.01$, represents a statistical difference. ns-not significant. Data shown in the graphs are mean ± SEM. n represents number of samples analyzed in the experiment, scale bar-50μm.

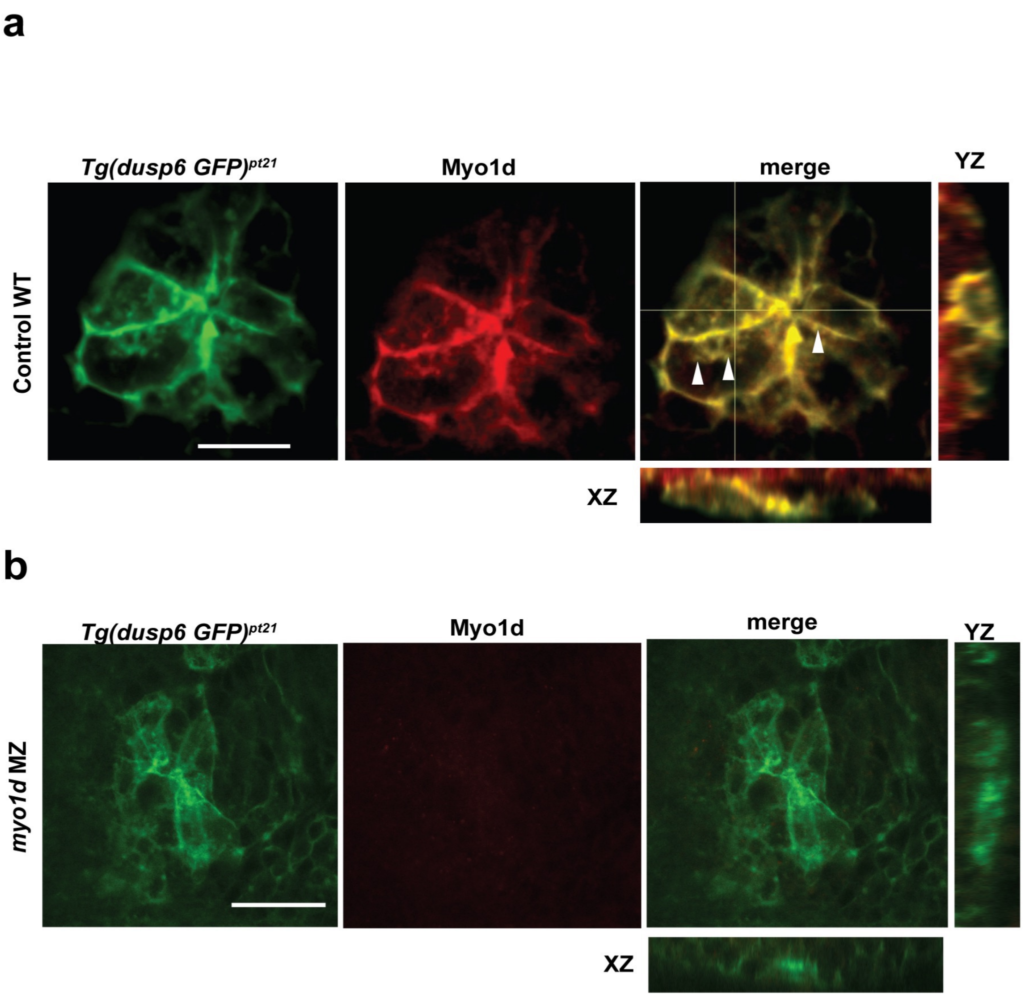

Supplementary Figure 2: Myo1D expression in KV epithelial cells colocalizes with vacuoles and are absent in *myo1d* MZ mutants.

a. *Tg(dusp6:GFP-MA)* embryos stained with Myo1D antibody (red) shows co-localized expression of Myo1D to membranes, and punctate structures in KV epithelial cells at 1-2 S.

b. Myo1d staining in *myo1d* MZ embryos shows loss of Myo1D expression, suggesting the *pt31a* allele is a null mutant (scale bar-50μm).

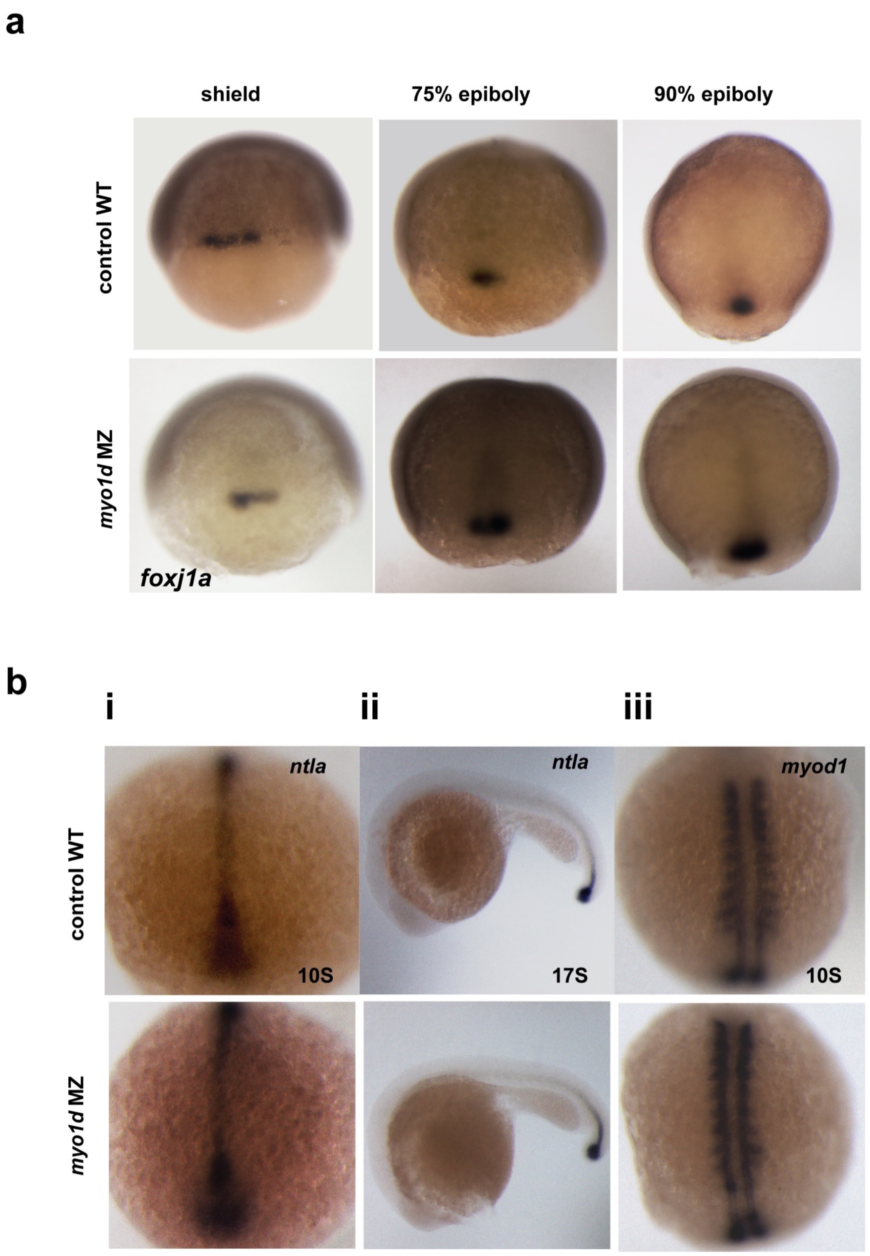

Supplementary Figure 3. Dorsal Forerunner Cells (DFC) clustering and migration or midline formation is normal in *myo1d* MZ mutant embryos

a. DFC clustering and migration in wildtype and *myo1d* MZ embryos analyzed by *foxj1a* expression at 60% epiboly, 75% epiboly, 90% epiboly (total n=195).

b. *tbxta* expression at (i) 10S, n=21 (WT), n=27 (*myo1d* MZ) (ii) 17s n=26 (WT), n=29 (*myo1d* MZ) showed normal midline expression in *myo1d* MZ mutants. *myod1* (*myogenic differentiation 1*) expression at 10S n=29 (WT), n=34 (*myo1d* MZ) was normal in somites and midline adaxial cells in *myo1d* MZ embryos.

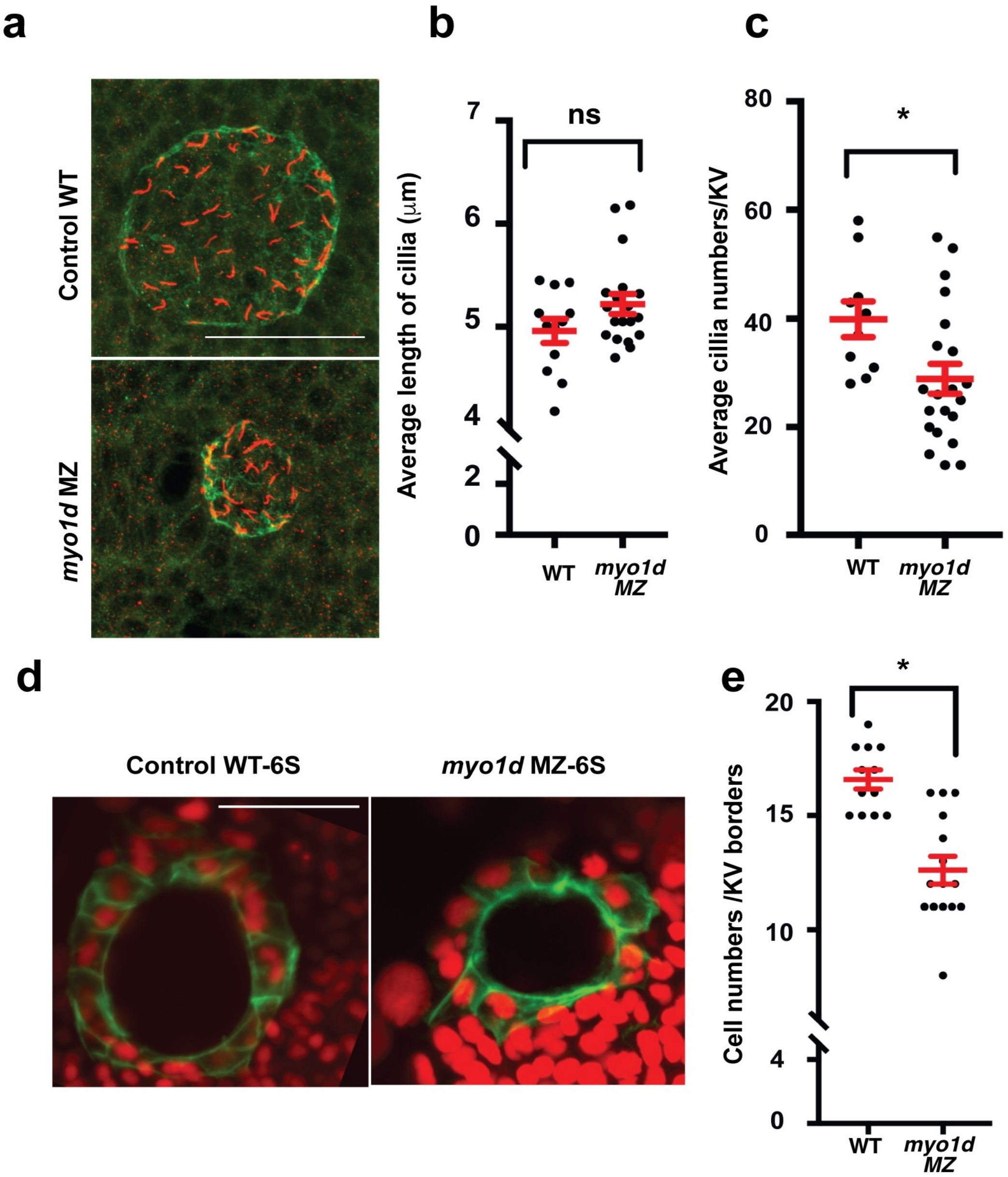

Supplementary Figure 4. Ciliogenesis appeared unaffected in zebrafish KV

a. Kupffer's vesicle at 8 S embryos stained for aPKC (green) and acetylated tubulin (red). Cilia appeared crowded in *myo1d* MZ embryos compared to controls.
b. Average cilia length of KV were not significantly affected (n=31).
c. Average cilia numbers of KV were significantly less (n=31).
d. *Tg(dusp6:GFP-MA)* embryos injected with *H2BmCherry* mRNA showing KV epithelial cell morphology. In this experiment, number of nuclei bordering KV epithelial cell borders were counted.
e. Cell numbers lining KV borders at 6S significantly affected in *myo1d MZ* mutants. (n=28).

Two sample comparisons in all the graphs were from unpaired t test. Data shown are mean ± SEM. * $p<0.05$ represents a statistical difference. ns-not significant. Scale bar-50μm.

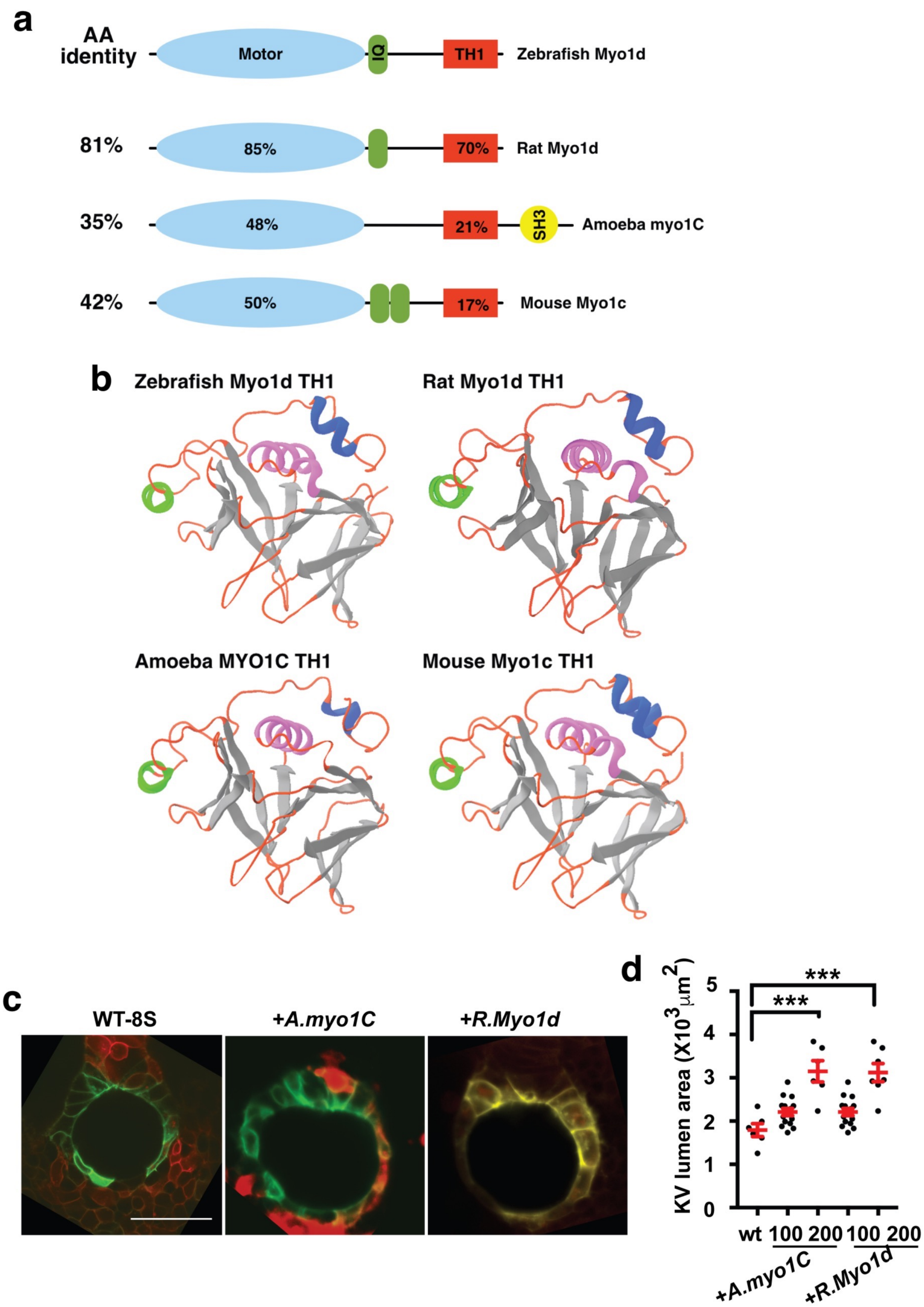

Supplementary Figure 5: Amino acid comparison of myosin1 showing protein domains.

a. Class I myosins showing myosin motor (blue), calmodulin binding IQ motif (green), Tail Homology 1 (TH1, red) and SH3 (yellow) domains. Total amino acid identity is listed on the left column compared to zebrafish Myo1d. Amino acid identity to zebrafish Myo1D motor and TH1 motifs are listed within the domains.

b. Images of the predicted TH1 domains showing similarity after Phyre2 modeling.

c. Representative images of KVs from *Tg(dusp6:GFP-MA)* embryos injected with *H2BmCherry* mRNA and *A. myo1C*, or Rat *Myo1d* showing expansion of the KV lumen area.

d. Graph showing KV lumen area after injection of various doses of myosin I family. Statistical comparisons for the graphs were by one-way ANOVA and post hoc analysis with Turkey's multiple range tests. *** $p<0.001$ represents a statistical difference Data shown in the graphs are mean ± SEM.

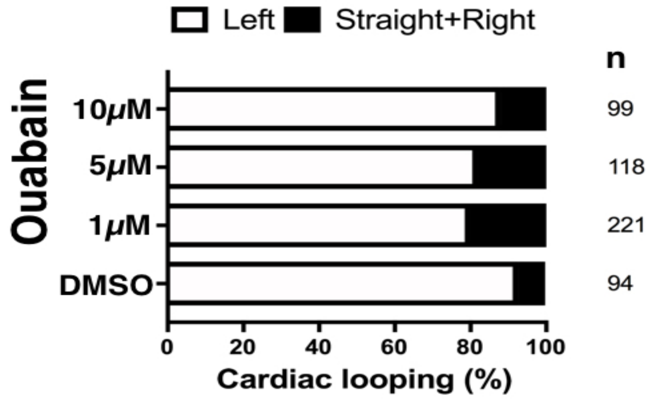

Supplementary Figure 6. Ouabain treatment at bud to 8S results in cardiac looping defects.

Increasing doses of ouabain treatment in WT zebrafish embryos from bud stage to 8S results in laterality defects.
