# Supplementary material for: Vertebrate myosin 1d regulates left–right organizer morphogenesis and laterality
Source: Nat Commun. 2018 Aug 23;9:3381. doi: 10.1038/s41467-018-05866-2 (PMC6107537; doi:10.1038/s41467-018-05866-2)
Supplement: Supplementary file 3 — Description of Additional Supplementary Files [file 41467_2018_5866_MOESM3_ESM.pdf]

Description of Additional Supplementary Files

File Name: Supplementary Movie 1
Description: Time lapse representative movie showing lumen expansion from 3-8 S stage in *Tg(dusp6:GFP-MA)* embryo.

File Name: Supplementary Movie 2
Description: Time lapse representative movie showing lumen expansion at 3-8 S stage in *myo1d* MO injected into *Tg(dusp6:GFP-MA)* embryo.

File Name: Supplementary Movie 3
Description: Time lapse representative movie showing FITC fluorescent microbead movement in wildtype control at 8S. DIC images merged with FITC channel.

File Name: Supplementary Movie 4
Description: Time lapse representative movie showing FITC fluorescent microbead movement in *myo1d* MZ embryo at 8S. DIC images merged with FITC channel.

File Name: Supplementary Movie 5
Description: Confocal movie showing vacuolar transport in KV epithelial cells from a *Tg(dusp6:GFP-MA)* embryo at 3S stage.

File Name: Supplementary Movie 6
Description: Confocal movie showing vacuolar transport in KV epithelial cells from a *myo1d* MZ;*Tg(dusp6:GFP-MA)* embryo at 3S stage.

File Name: Supplementary Movie 7
Description: Representative 4D STED movie showing vacuolar transport across KV epithelial cells in a *Tg(dusp6:GFP-MA)* embryo. L-lumen

File Name: Supplementary Movie 8
Description: Representative 4D STED movie showing vacuolar transport across KV epithelial cells in *myo1d* MZ*;Tg(dusp6:GFP-MA)* embryo. Note vacuolar size is larger than in wildtype embryo show in Supplementary Movie 7. L-lumen

File Name: Supplementary Movie 9
Description: 3D reconstructed movie showing posterior KV epithelial cells between 2-3 S stage in a *Tg(dusp6:GFP-MA)* embryo.

File Name: Supplementary Movie 10
Description: 3D reconstructed movie showing presence of large vacuoles in posterior KV epithelial cells at 2-3 S stage in *myo1d* MZ;*Tg(dusp6:GFP-MA)* embryos.
